# Ocular Pressure-Volume Relationship and Ganglion Cell Death in Glaucoma

**DOI:** 10.21926/obm.neurobiol.2102098

**Published:** 2021-05-20

**Authors:** Ji-Jie Pang, Samuel M. Wu

**Affiliations:** Department of Ophthalmology, Baylor College of Medicine, One Baylor Plaza, NC 205, Houston, Texas;

**Keywords:** Ocular pressure-volume, ganglion cell death, glaucoma

## Abstract

We studied how GC death in glaucoma related to the intraocular pressure (IOP), eyeball volume (V_S_) and elasticity (volumetric K_S_ and tensile E_S_), and eyeball volume-pressure relation. Glaucomatous GC loss was studied in DBA/2J (D2) mice with wild-type mice as controls. GCs were retrogradely identified and observed with a confocal microscope. The elasticity calculation was also done on published data from patients treated by a gas bubble injection in the vitreous cavity. The GC population in D2 mice (1.5- to 14-month-old) was negatively correlated with following factors: V_S_ (p = 0.0003), age (p = 0.0026) and IOP (but p = 0.0966). As indicated by average values, adult D2 mice (≥6 months) suffered significant GC loss, low K_S_ and E_S_, and universal expansion of V_S_ with normal IOP. K_S_ and E_S_ in the patients were also lower upon prolonged eyeball expansion compared to acute expansion. Based on the results and presumptions of a closed and continuous eyeball space (thereby ΔV_S_ ≈ ΔV_W_, ΔV_W_-the change in the aqueous humor amount), we deduced equations on the ocular volume-pressure relationship: ΔIOP = K_S_*ΔV_W_/V_S_ or ΔIOP = (2/3)*[1/(1-ν)]*(H/R)*E_S_*ΔV_W_/V_S_ (ν, Poisson’s ratio taken as 0.5; R, the curvature radius; and H, the shell thickness). Under normal atmospheric pressure, IOP of 10~50 mmHg contributed only 1.2~6.6% of the pressure opposing the retina and eyeball shell. We conclude: 1) A disturbance of ocular volume-pressure homeostasis, mediated primarily by low K_S_ and E_S_, expanded V_S_, and large ΔV_W_, is correlated with GC death in glaucoma and 2) D2 mice with GC loss and normal IOP may serve as animal models for human normal-tension glaucoma.

## 1. Description of the Paper

We revealed a perturbation of ocular pressure-volume homeostasis (low elasticity, eyeball expansion and the accumulation of aqueous humor) correlated with GC death in normal-tension glaucoma mice. Ocular pressure-volume relation was addressed for the first time by modification of bulk and Young’s modulus.

## 2. Introduction

Glaucoma is a serious blinding ocular disease, which is characterized by retinal ganglion cell (GC) death [1–8]. It has been known as unpreventable and incurable, because vision loss is often the early detectable symptom and neurons usually do not regenerate. It is very important to treat the disease before GCs die, but due to limited understanding of the exact mechanism of GC death, currently there is no effective approach for diagnosis and treatment of the disease in its subclinical stage [9, 10].

Glaucoma usually affects one eye earlier and more severely than the other. Sometimes it affects only one eye, especially in patients with so-called normal or low-tension glaucoma (NTG or LTG) [11]. Age, race and genetics are some known risk factors for glaucoma [12, 13]; but they are less accountable for monocular cases of the disease. Intraocular pressure (IOP) is a long-known risk factor for glaucoma; However, GC death is not always associated with elevated IOP [14]. Some patients may have ocular hypertension without vision loss; and vision loss may occur for patients with NTG. Von Graefe described NTG condition as early as 1857. It consists of typical glaucomatous disc and field changes, an open angle and pressures within the statistically normal range. So far, fewer known mechanisms may clearly explain vision loss in NTG [11, 15]. IOP asymmetry in patients with NTG is reported to be unrelated to visual field asymmetry [16]. NTG raised a fundamental question regarding the causal relationship between pressure and the disc and field changes. Ischemia was reported to be responsible for the optic nerve damage in NTG patients who suffered migraine [17], shock, blood loss, low blood pressure and optic disc hemorrhages [18]. Yet, the data on ocular blood flow in NTG are still highly conflicting [15, 19].

The circulation of the aqueous humor has been studied previously, and many critical data have been obtained on normal and glaucoma patients [20]. A quantitative relation between IOP level and the amount of aqueous humor is still absent, however, which leaves it a question whether IOP is solely dependent on aqueous humor. Additionally, it is possible that the physical interaction between the eyeball shell and the eyeball contents also influence IOP. This interaction is important for maintaining the eyeball’s physical homeostasis, but how it relates to glaucoma [7, 13, 19, 21–24] is still unknown.

The behavior of spherical shells has been an important topic in physics and mathematics. The eyeball wall resembles a closed spherical shell; and its behavior is not clearly understood in glaucoma. The eyeball shell is elastic and the tensile elasticity of the sclera, cornea and choroidal complex have been studied previously in vivo or on tissue strips in the human and pig [25–27], and it ranges from 2.45 ×10^4^ to 2.9 ×10^6^ N/m^2^. For elastic materials, the elasticity largely determines the relationship between force and length or pressure and volume. The elastic eyeball shell is constantly exposed to IOP and atmospheric pressure (ATM). Thus, the retina, a thin layer of soft neural tissue attached to the inside of the eyeball shell, is inevitably subjected to changes in the eyeball’s physical environment. However, despite the great attention on IOP in glaucoma studies, most physical properties of the eyeball (e.g. the volumetric elasticity of the eyeball-K_S_, tensile elasticity of the shell-E_S_, eyeball volume-V_S_ and the relation among IOP, K_S_, E_S_, V_S_ and the volume of aqueous humor (V_W_)) have not been previously examined in glaucoma. Consequently, it is unclear what role they play in GC death in glaucoma. The current report intends to fill this blank.

## 2. Materials and Methods

### 2.1 Animals

The animals used in this study were DBA/2J (D2) and C57BL/6J (B6) mice purchased from Jackson Laboratory (Bar Harbor, ME, USA). The D2 mouse develops glaucoma associated with iris stromal atrophy and iris pigment dispersion phenotypes. Genetic studies defined two separate loci that contribute to the overall phenotype in the DBA/2J mouse, ipd and isa. Either mutations in a homozygous state contributes to glaucoma. The mice were 1.5- to 14-month-old males and females. All procedures used in this study followed the NIH and ARVO animal care guidelines as well as the relevant requirements of the Baylor College of Medicine Animal Care and Use Committee. All mice were dark-adapted for 1~2 hours prior to the experiment. Animals were anesthetized with an intra-peritoneal injection of ketamine (200 mg/kg) and xylazine (10 mg/kg). The eyes were enucleated after animals were deeply anesthetized. Animals were sacrificed by over-dose of the anesthesia thereafter.

The mice were divided into two experimental groups by age, the young group <6 months and adult group ≥6 months. We randomly selected healthy mice at desired ages for the experiment without IOP preference. IOP was routinely measured with a tonometer in the deep anesthetized condition before enucleation. It was classified as normal (<13 mmHg), moderately high (13 to 16 mmHg) and high (>16 mmHg). The pathological alterations in adult D2 mice were evaluated by comparing to those in young D2 mice, while those of young D2 mice were evaluated by comparing to age-matched young wild-type mice. The adult wild-type mouse was not used as control for the adult D2 mouse, considering that they can develop age-related disease, including glaucoma, as the adult D2 mouse.

### 2.2 Retrograde Labeling of GCs and Immunocytological Staining

Freshly dissected whole retinas were used for retrograde labeling. Previously established techniques were precisely followed [28]. Briefly, a mixture of neurobiotin, a gap-junction-permeable dye (NB, MW 322.85, Vector Laboratories, CA), and Lucifer yellow, a less permeable dye (LY, MW 457.24, Sigma, MO) [29–31], were used for the labeling. Eyeballs with an attached optic nerve stump were chosen for retrograde labeling. First, the nerve stump was dipped into a small drop (3μl) of a cocktail that contained 3% LY and 8% NB in the internal solution [32] for 20 minutes. Afterwards, the eyeball was thoroughly rinsed with oxygenated Ames’ medium (Sigma) to remove the extra dye. Then the eyeball was dissected under infrared illumination. The eyecup with intact retina and sclera tissue was transferred into fresh oxygenated Ames’ medium and kept at room temperature for 40 minutes under a 10 min-dark/10 min-light cycle. The medium that retinas were incubated in was replaced every few minutes by fresh medium during the labeling. Following the light cycle, the whole retinas were rinsed and fixed in darkness in 4% paraformaldehyde (Electron Microscopy Sciences, PA) and 0.05 % glutaraldehyde (Sigma) in phosphate buffer (D-PBS, Invitrogen, CA), pH 7.4, for 30–45 min in room temperature. The retinas were blocked with 10% donkey serum (Jackson Immunoresearch) in TBS (D-PBS with 0.5% Triton X-100 (Sigma) and 0.1% NaN_3_ (Sigma)) for 2 hours at room temperature or at 4 °C overnight to reduce nonspecific labeling. Afterwards, the retrogradely filled whole retinas were incubated in Cy3 or Cy5-conjugated streptavidin (1:200, Jackson Immunoresearch, PA) in 3% normal donkey serum-TBS for 1 day at 4 °C.

Some retinas were subsequently cut into 40 μ m-thick vertical sections with a vibratome. The whole-mounted retinas or free-floating sections were incubated in primary antibodies in the presence of 3% donkey serum-TBS for 3–5 days at 4 °C. Controls lacking primary antibodies were also processed. Following several rinses, the slices and whole retinas were then transferred into Cy3- and/or Cy5- conjugated secondary antibodies (1:200, Jackson Immunoresearch) and/or Alexa Fluor 488-conjugated secondary antibodies (1:200, Molecular Probes, CA), in 3% normal donkey serum-TBS solution in 4 °C overnight. After extensive rinses, the slices and whole retinas were coverslipped. Two small pieces of filter paper (180 μm thick, MF-membrane filters, Millipore, MA, USA) were mounted beside whole retinas to prevent them from being over-flattened. A fluorescent nuclear dye, TO-PRO-3 (1: 3000, Molecular probes, Eugene, OR) was used to visualize nuclei in retinas. It was used together with secondary antibodies.

**Figure 1:**
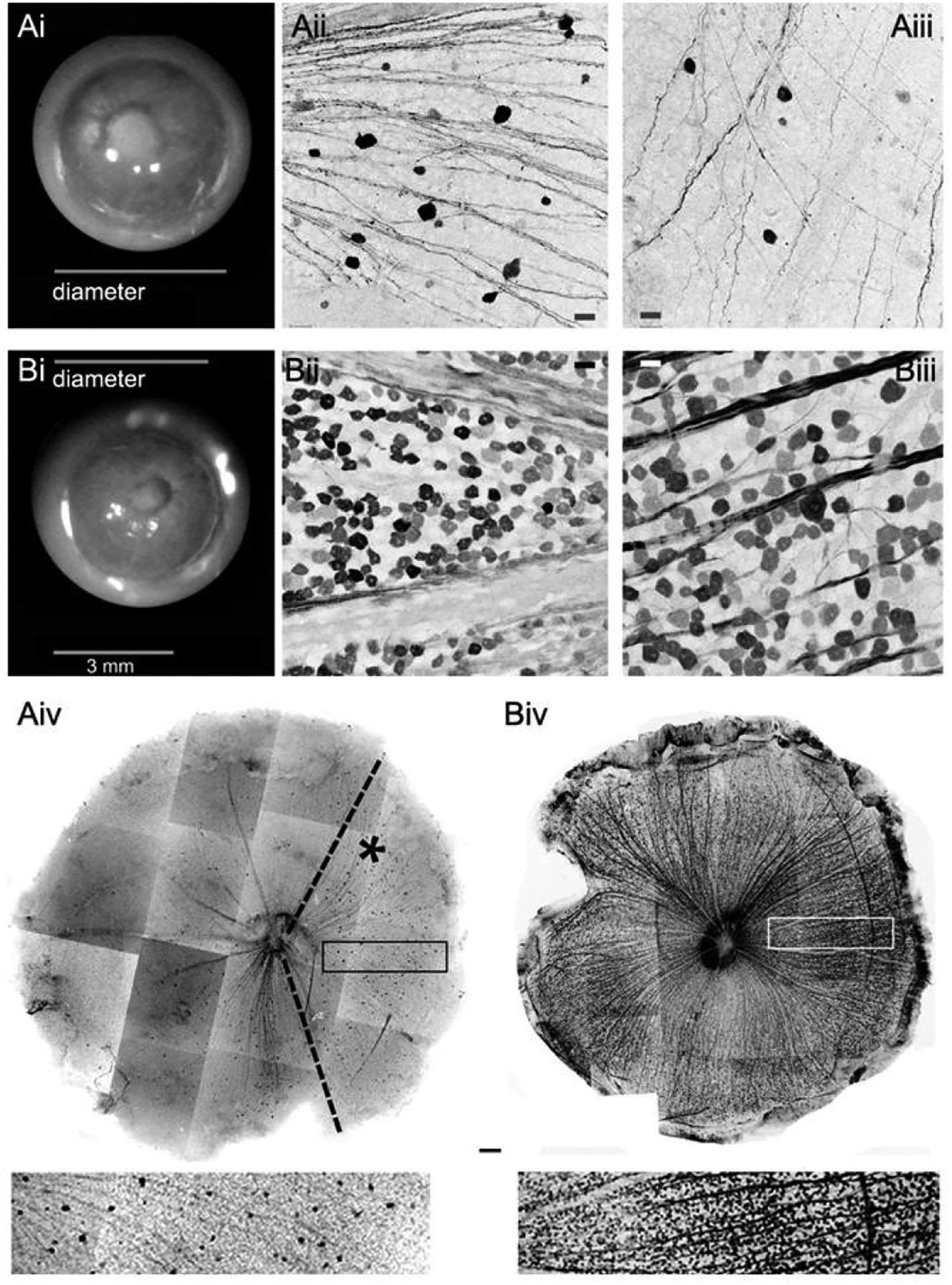
Eyeball expansion and GC loss in the adult DBA/2J mouse retina. Front-view images of freshly dissected intact eyeballs at the coronal plane were taken under a dissecting microscope (i), in which the bright background surrounds the eyeballs. The two eyeballs belong to the same mouse (A-left and B-right). The left eye has a deformed pupil and a larger volume (Ai); and the right eye has a smaller volume (Bi). Both eyeballs possess a large cornea. Retinas were retrogradely labeled by Lucifer yellow and neurobiotin (black). Confocal micrographs focused on the GCL are taken from the whole mounted retinas, including the central retina (ii) and the peripheral retina (iii). Whole retinal images (iv) were composed from individual confocal micrographs with Photoshop software. GC density in the smaller eyeball is nearly normal (Biv). GCs in the larger eyeball are largely lost (Aiv); yet in a large fan-shaped region (asterisk) GCs maintain a low density that is nearly even from the central to the peripheral retina (insert). This indicates that GC loss in glaucoma is related to eyeball volume, and some subtypes of GCs are less vulnerable in glaucoma. GC-ganglion cell; GCL-GC layer; Scale bar: 3 mm in i, 20 μm in ii and iii and 500 μm in iv.

The preparations were observed with a laser scanning confocal microscope (LSM 510, Carl Zeiss, Germany). Images were further processed in Adobe Photoshop v9.0.2. For better clarity, some images were presented in black and white, in which fluorescent signals were in black against a bright background (Figure 1ii–1iv).

### 2.3 Data Analysis

All data are presented as mean ± standard error of the mean. The difference between data groups was analyzed by two-tail student *t*-test. Correlations among data groups were analyzed with Microsoft Excel 2000 and Sigma Plot 11.2. K_S_ was estimated by volumetric stress versus volumetric strain (bulk modulus) [33]:

(1) $$K_{S}=\Delta IOP/\left(\Delta V_{s}/V_{s}\right)$$

where ΔIOP (N/m^2^) and ΔV_S_ were calculated as current measurements minus the minimum values observed, which were 16.5 μl for V_S_ and 6.5 mmHg for IOP in the D2 mouse and 17.1 μl and 7 mmHg in the wild-type mouse. K_S_ was used, assuming the aqueous humor volume was fully adjusted and stable. K_S_ is theoretically primarily determined by the tensile, shear, bulk modulus of the eyeball shell while the bulk modulus of water and the eyeball content is constant, yet the relationship of these variables in a closed thin-wall shell is still absent to our best knowledge. Thus, K_S_ calculation here was simplified by taking the eyeball as a single unit. E_S_ calculation refers to a previous equation [25]:

(2) $$E_{s}=(3/2)*(1-v)*(R/H)*\Delta IOP/\left(\Delta V_{s}/V_{s}\right)=0.75*(R/H)*\Delta IOP/\left(\Delta V_{s}/V_{s}\right)$$

where Poisson’s ratio (ν) is taken as 0.5 [25], R is the curvature radius, and H is the shell thickness

K_S_ and E_S_ are also calculated by using the difference of the average IOP of the young and adult mouse (as ΔIOP) and the difference of average V_S_ (as ΔV_S_) in Equation (1) and (2), which are termed K_SM_ and E_SM_, respectively.

V_S_ was measured in two ways, either by emerging them into a graduated tube filled with Ames medium and reading the eyeball volume directly, or by measuring eyeballs in photos and calculating their volume with the following equation:

(3) $$V_{s}=(4/3)*\pi *R_{o}^{3}$$

(4) $$R_{o}={\left[\left(3*V_{s}\right)/(4\pi )\right]}^{1/3}$$

where R_o_ is the outer radius of the eyeball. The thickness of the eyeball shell is termed H (adopted 50 μm for the wild-type mouse and 33μm for the D2 mouse) [34]. Hence, the inner surface radius (R_i_) of the eyeball is:

(5) $$R_{i}=R_{o}-H$$

The anterior of the eye is covered by the cornea. The retina lines the inner surface of the posterior portion of the eyeball. Given the height of the spherical cap of the cornea (Z_C_), the height of the spherical cap that retina covers (Z_R_), the depth of the eyeball (d_z_, = 2R_o_), H<<d_z_, then the coverage of the retina in the inner surface of the eyeball can be described by α:

(6) $$\alpha \approx Z_{R}/d_{z}\approx \left(d_{z}-Z_{C}\right)/d_{z}=1-Z_{C}/d_{z}$$

and the coverage of the cornea can be estimated by β:

(7) $$\beta \approx Z_{C}/d_{z}\approx 1-\alpha$$

The volume of the cornea spherical cap (VC) is calculated for estimation of the space of the anterior chamber:

(8) $$V_{C}=(1/3)*\pi *{\left(Z_{C}-H\right)}^{2}*\left(3R_{i}-Z_{C}+H\right)$$

where (Z_C_-H) represents the inner height of the cornea spherical cap. In the GCL, GCs are usually arranged in a single layer. The total number of GCs were obtained either by counting all GCs or by appropriate sampling from peripheral and central retina [28]. The total retinal area was directly measured on whole retina images composed by individual confocal micrographs with Photoshop software.

## 3. Results

**Figure 2:**
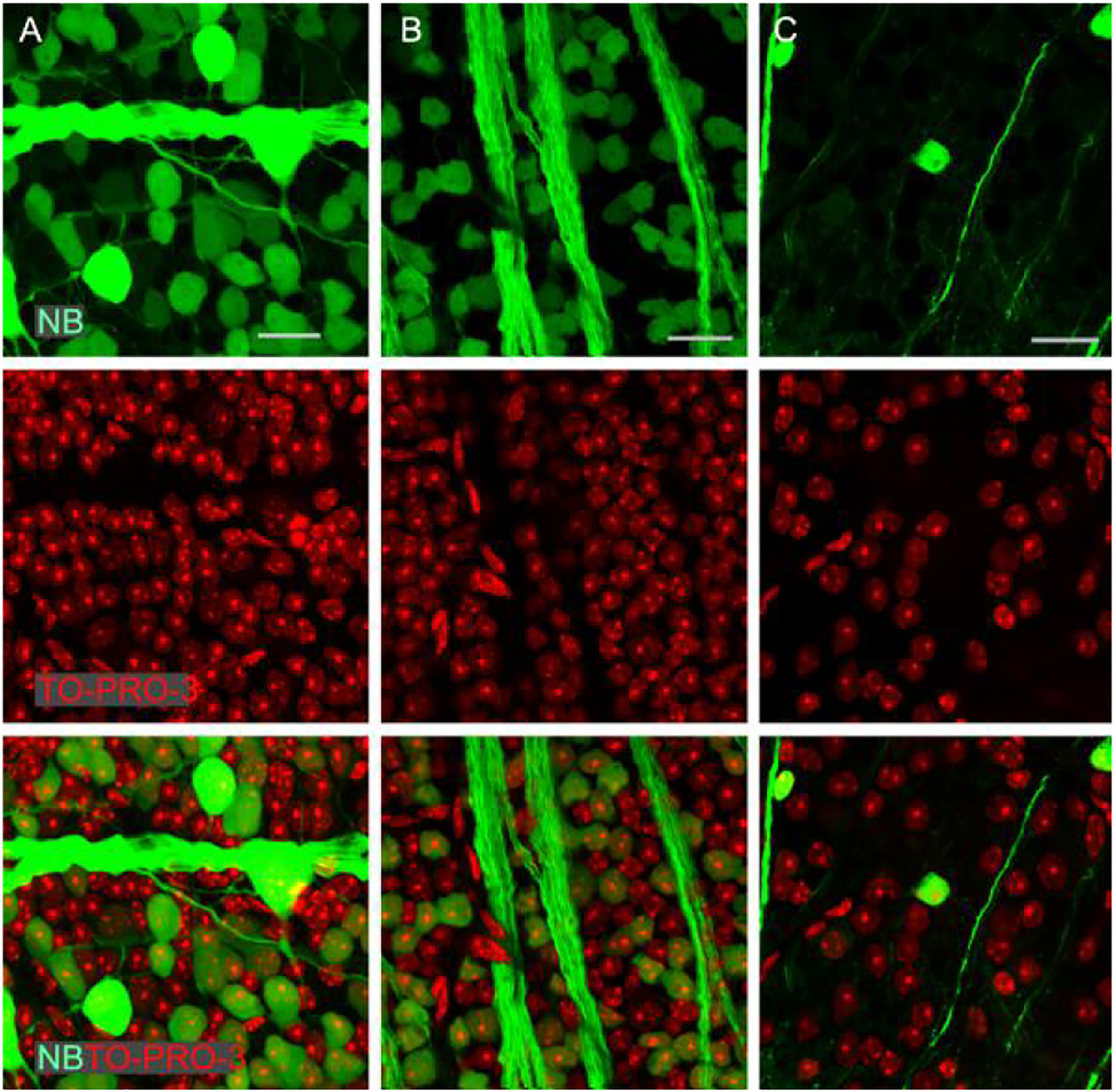
Loss of GCs and total neurons in the GCL in the adult D2 mouse (C) compared with the young D2 mouse (B) and young wild-type B6 mouse (A). Confocal micrographs from flat-mounted retinas are retrogradely labeled by NB for GCs (green, upper panels) and stained by TO-PRO-3 for the nuclei of all neurons (red, middle panels). Bottom panels: merged images of the red and green channels. A: A retina from a 5-month-old B6 mouse with normal IOP. B: A retina from a 4-month-old D2 mouse with normal IOP. The retinas in A and B have a similar density of GCs and total neurons in the GCL. C: A retina from a 10-month-old D2 mouse with eyeball expansion and normal IOP, where retrogradely labeled GCs and axon bundles are largely diminished and TO-PRO-3 reveals fewer neurons in the GCL. B6: C57BL/6J; D2: DBA/2J; normal IOP: <13 mmHg; expanded eyeball: volume >30 μl; GC: ganglion cell; GCL: GC layer; NB: neurobiotin; IOP: intraocular pressure; scale bar for all panels: 20 μm.

We first characterized physical properties of the eyeball and retina for quantifying the physical disturbance. Then, we investigated the relationship between the retinal pathology and the physical disturbance in D2 mice and further compared D2 mice with wild-type mice to determine how the presence and absence of physical disturbance affected the retinal pathology in D2 mice. As indicated by average values, adult DBA/2J mice suffered significant GC loss, low K_S_ and E_S_ and large V_S_ with normal IOP (Figure 1 and Figure 2). Vs expanded homogeneously. The GC population was negatively correlated with following factors: V_S_ (*p* = 0.0003), age (*p* = 0.0026) and IOP (but *p* = 0.0966). Wild-type mice, on the other hand, showed different physical properties without RGC loss. The results are detailed in the following sections.

### 3.1 Retrogradely Identified GCs and Total Neurons in the GCL were Reduced in the Adult D2 Mouse and Negatively Correlated with the Eyeball Depth (d_z_), Width (d_x_) and Height (d_y_), V_S_, and Age

We used retrograde labeling for identification of retinal GCs [28]. Retrogradely labeled retinas were further stained with the nuclear dye TO-PRO-3 to reveal total neurons in the GCL. GCs and total neurons were counted in the GC soma plane, where nuclei of Müller cells and astrocytes were not present [28]. The TO-PRO-3 stained nuclei, excluding irregular-shaped intensively stained nuclei of microglial cells and endothelial cell nuclei of retinal blood vessels [28], were counted as total neurons. Retinal GCs were usually evenly labeled over the entire retina, but sometimes GC somas in the peripheral retinal were labeled more brightly than those in the central retina probably due to their large soma size and presumably thicker axons. In the D2 mouse, GC density was often reduced together with the density of TO-PRO-3-labeled nuclei (Figure 2), which indicated a real GC loss. The number of GCs was calculated separately from healthy and damaged retinal areas by GC density * the area size.

**Figure 3:**
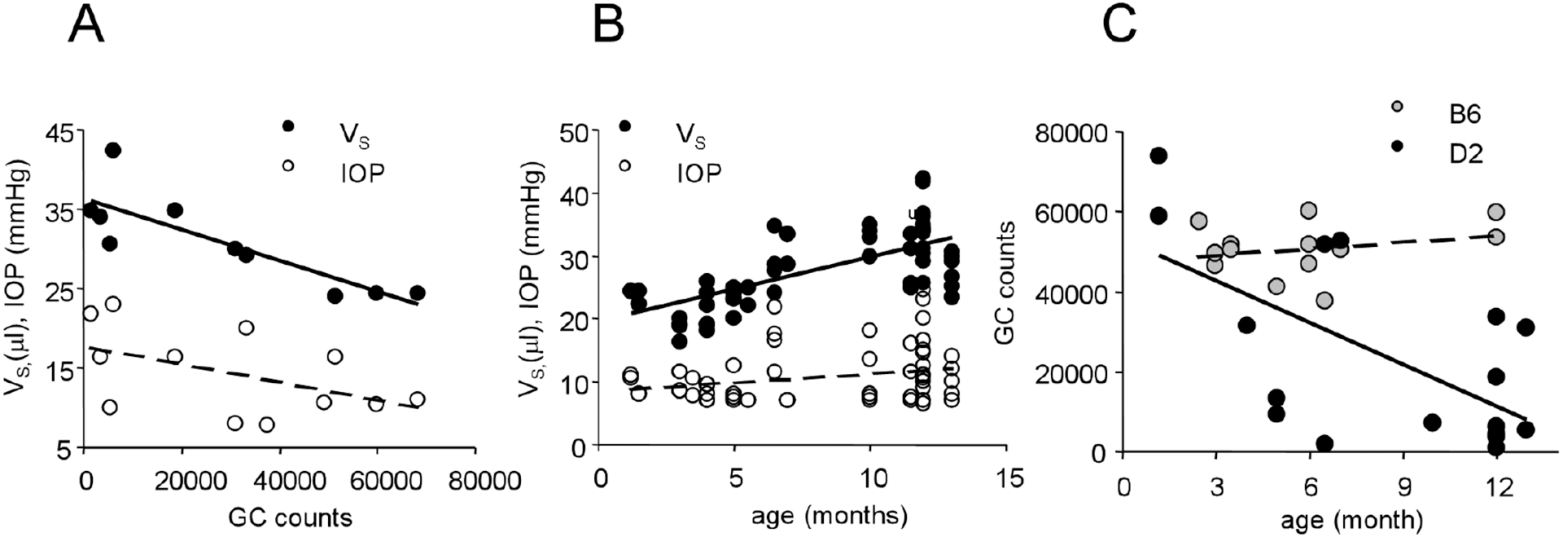
GC population is negatively correlated to Vs in the D2 mouse retina. Scatter plots show a negative correlation between GC counts with Vs (*p*<0.001) or IOP (but *p* = 0.096) (A), age-correlated increase of Vs (*p*<0.001) and IOP (*p* = 0.045) (B) and age-correlated reduction of GC population (*p* = 0.005) in the D2 mouse (C). GC counts do not significantly change in the wild-type mouse (C). Vs-eyeball volume; IOP-intraocular pressure; B6-C57BL/6J; D2-DBA/2J.

In the wild-type mouse (3~14 months), in agreement with our previous report [28], the GC population was ranged between 40000 and 60000 cells (n = 13), averaging 50420 ± 1825 cells per retina. The total neurons in the GCL ranged from 105,000 to 125,000 cells per retina, averaging 111991 ± 2513 cells. GCs were nearly 44.4% ± 1.8% of the total neurons in the GCL. Within the observed life span, the total number of retrogradely labeled GCs and the number of total neurons did not change with age for the wild-type mouse (Figure 3).

In the D2 mouse, the neuron populations (GCs and total neurons) in the GCL were *negatively* correlated with following factors (in the order of statistical significance of the correlation coefficient): d_z_>Z_C_>d_x_/d_y_>V_S_>age>IOP (Figure 1, Figure 3, Table 1A, Table 1B, Table 2, and Table 3). By contrast, the GC population was *positively* correlated with IOP (*p* = 0.025) and V_S_ (*p* = 0.047) in the wildtype mouse. This suggested a harmful passive expansion of the eyeball and retina in the adult D2 mouse and normal growth in the wild-type mouse.

In the adult D2 mouse, the GC population and the total neurons in the GCL were largely reduced, although the extent varied among individual mice. In young D2 mice, neuron populations were close to those in wild type mice. Displaced ACs in the GCL, estimated by subtracing GCs from total neurons in the GCL, was significantly reduced in the adult D2 mouse (Figure 2 and Table 1).

GC loss in the D2 mouse usually presented as irregular areas with fewer or no GCs. In young D2 mice, such areas were usually small and observed frequently in the peripheral retina. In adult D2 mouse retinas, damaged areas were larger. Between 4 and 9 months of age, damaged areas might cover fan-shaped sectors, half of the retina, or the entire retina. At around 1 year of age some retinas were nearly absent of any GCs and axonal bundles. See Figure 1.

### 3.2 Significant Eyeball Expansion was Observed in Adult D2 Mice with Normal IOP

The average IOP in adult D2 mice was not significantly different from the wild-type mice, though IOP in the young D2 mice was lower (Table 1). IOP in wild-type mice did not clearly change with age (*p* = 0.237, n = 30). An age-related increase was observed in IOP (*p* = 0.045 and n = 64) and V_S_ (*p*<0.0001, n = 57) in the D2 mouse and V_S_ (*p*<0.0001, n = 18) in the wild-type mouse.

V_S_ was not correlated with IOP in the wild-type mouse (*p* = 0.223, n = 15); but V_S_ positively correlated to IOP in the D2 mouse (*p* = 0.007, n = 57). The average V_S_ in the adult D2 mouse was significantly larger compared with the young D2 mouse and the wild-type mouse (Figure 1, Figure 3 and Table 1). The average expansion rate, estimated by the difference of the average volume versus the difference of the average age between the young and the adult mouse, was 0.87 μl or 4% increase per month for the wild-type mouse and 1.91 μl or 9% increase per month for the D2 mouse. The moderate expansion in wild-type mice did not cause GC loss and thereby was recognized as physiological growth. This indicates that eyeball expansion below 4% per month might be acceptable or adaptable for retinal neurons and ocular tissue. However, extensive expansion, as seen in the adult D2 mouse, could be a serious challenge for normal visual function.

### 3.3 Eyeballs Possessed a Nearly Perfect Spherical Shape and Expanded Universally in Adult D2 Mice

**Figure 4:**
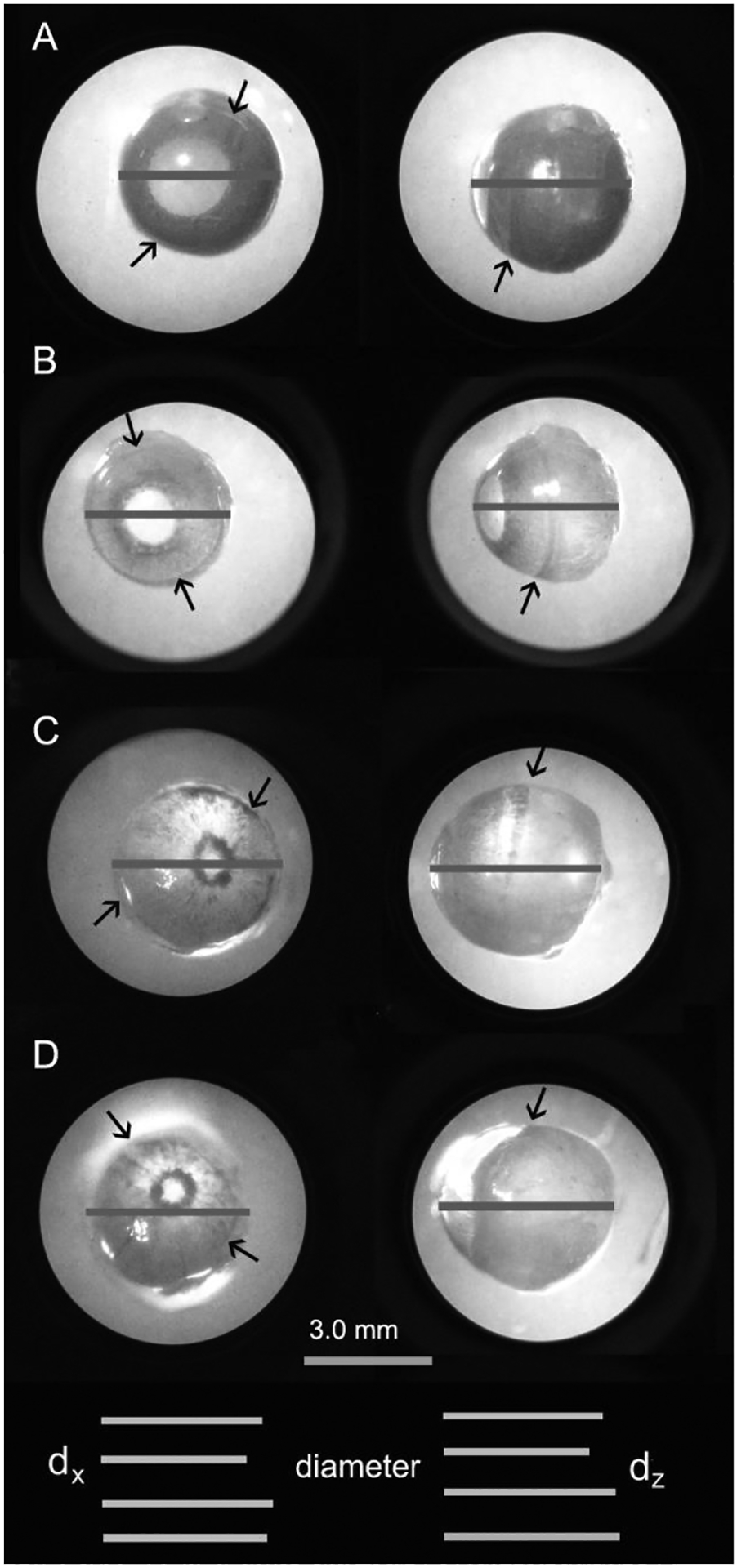
Eyeball dimensions in the wild-type and D2 mouse. Front-view (left panels) and side-view (right panels) images of eyeballs from the wild-type mouse (A, 5-month-old with normal IOP) and the D2 mouse (B, 4-month-old with normal IOP; C, 10-month-old with high IOP and eyeball expansion and D, 10-month-old with normal IOP and eyeball expansion) were taken under an infrared illuminated dissecting microscope. Eyeballs are encircled by the bright background illumination. Bars superimposed on the eyeballs denote their diameters. For better comparison, the bars are also listed together beneath the images with the same order, the width (d_x_) in the left and the depth (d_z_) in the right. The data indicates that the mouse eyeball possesses a nearly perfect spherical shape. Eyeballs in adult D2 mice (C and D) have a large volume, large cornea but smaller pupil. Arrows show the edge of the cornea, where a shallow indentation is visible in the young mouse but nearly disappeared in the adult D2 mouse. D2-DBA/2J; B6-C57BL/6J; normal IOP- <13 mmHg; high IOP- >16 mmHg; expanded eyeball-volume >30 μl.

**Figure 5:**
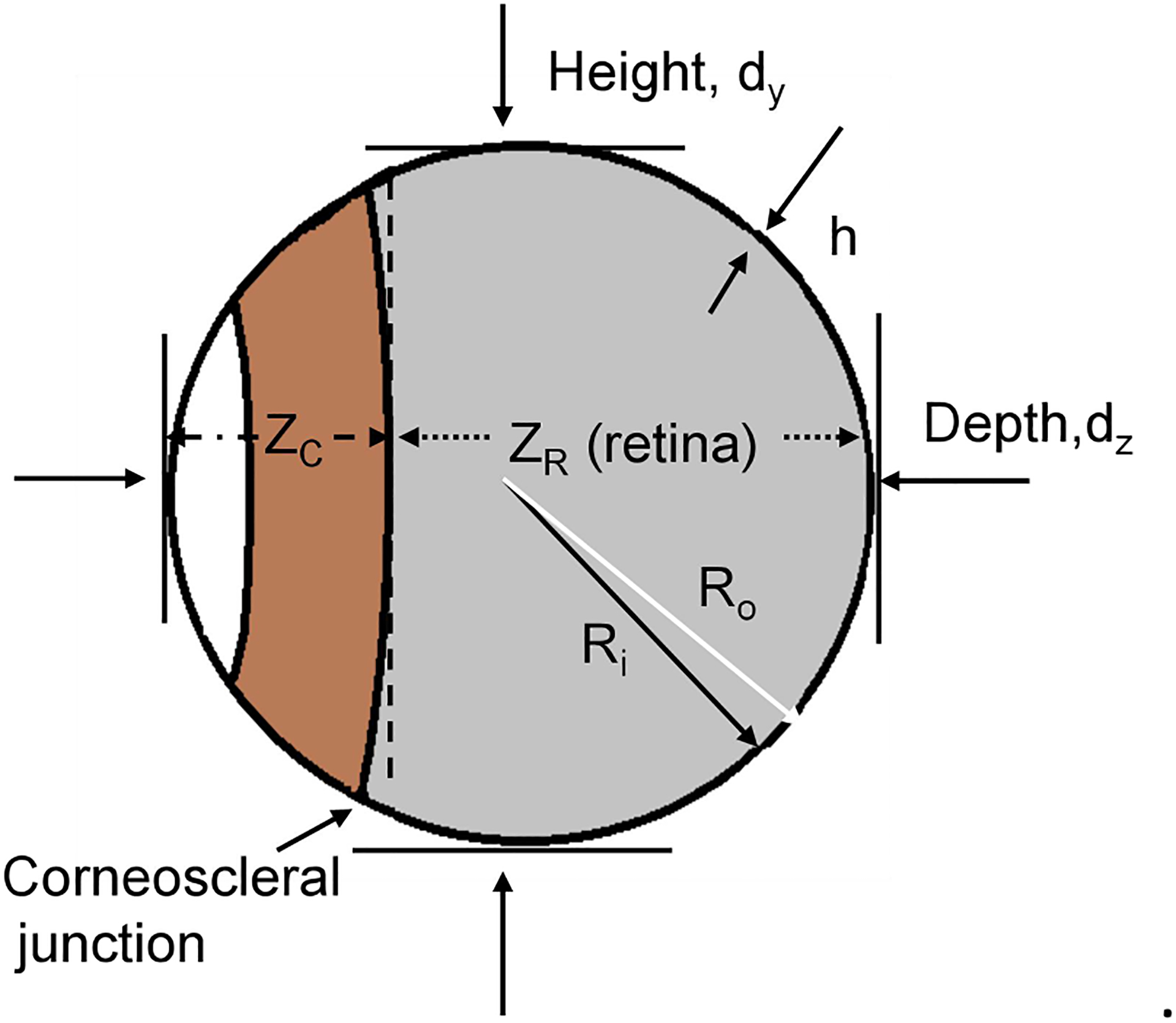
Terms for Eyeball measurements.

Eyeballs in the adult D2 mouse usually showed a small and irregular pupil, enlarged cornea area, larger anterior chamber angle, and iris depigmentation. To precisely measure eyeball volume and the elasticity, we studied the physical shape of eyeballs (Figure 4). We measured their volumes directly and/or on photos in vitro (direct measurement). Accurate front-view (coronal plane) and side-view pictures of eyeballs (sagittal plane) were taken under dissection microscope. For better Z_C_ measurement in side-view pictures, eyeballs were oriented in such a position so that the edge of the cornea looked like a straight line (Figure 4 and Figure 5).

The height, width and depth of the eyeball (d_x_, d_y_ and d_z_) in young D2 mice were not significantly different from those in wild type mice. They were significantly bigger in adult D2 mice, however (Table 2). The eyeballs in adult D2 mice were slightly elongated but did not show significant age-related progressive development. The d_x_: d_y_: d_z_ ratio calculated based on direct measurements in vitro was 1: 1: 1 in the wild-type mouse, 1: 1: 1.044 in the young D2 mouse and 1: 1: 1.005 in the adult D2 mouse. Z_C_ was slightly larger in the adult D2 mouse than the young D2 and wild-type mouse, but the difference was not statistically significant (*p* = 0.059 and 0.207, respectively) (Table 2). The data indicated that the eyeball was almost perfectly spherical in the mouse and that the eyeball expanded universally in the adult D2 mouse. It further suggested that the pressure inside the eyeball was nearly homogeneous.

Because of the spherical shape, the eyeball volume can also be estimated by measuring the arc of the anterior spherical cap. This is applicable on living animals by taking side-view pictures of the eye (non-invasive measurement). A circle overlapping the cap provides a radius for calculation of the eyeball volume. Given the height (H_C_) and the width (W_C_) of the arc, the radius of the circle or eyeball can be calculated by:

(9) $$R_{o}=H_{C}/2+W_{C}^{2}/\left(8H_{C}\right)$$

We used both direct and non-invasive approaches to measure eyeball size on some animals and compared the results. Due to the slight elongation of the eyeball in the adult D2 mouse, V_S_ estimated by non-invasive measurement was slightly smaller (0.5–4%) than that obtained from direct measurement. It indicated that the non-invasive approach was useful for revealing a V_S_ change of 5% or more. Since there was a shallow indentation at the sclerocorneal junction (though it was less obvious in adult D2 mice than in the young ones), to get a better result from noninvasive measurement, the side-view images of the eyeballs require to expose the anterior eye beyond the cornea. This non-invasive approach is potentially applicable in human patients.

The side-view images of the eyeballs were also used to examine the height of the cornea spherical cap (Z_C_) in order to estimate the space of the anterior chamber and the coverage of retina. The β value (average Z_C_/average d_z_) was 38% in the wild-type mouse, 35% in the young D2 mouse and 36% in the adult D2 mouse, and it was not correlated with age. Similarly, the α value (1-Z_C_)/d_z_ ratio was not significantly different between the D2 (near 65%) and the wild-type mouse (62%). The data suggested that the eyeball enlargement in the adult D2 mouse was nearly proportional and caused nearly universal enlargement of the eyeball, the anterior chamber, and the retinal area.

Additionally, using the average Z_C_ and R_i_ in Equation (8), the anterior chamber space (including the space occupied by iris, lens and ciliary body) was calculated as 6.6μl in the wild-type mouse, which is nearly 20% larger than the total volume of the aqueous humor directly measured in wild-type mice (4~6μl, n = 4). The chamber spaces were estimated to be 5.8μl and 8.8μl for the young and adult D2 mouse, respectively.

### 3.4 Lower Eyeball Elasticity in D2 Mice Resembled Human Patients with Prolonged Eyeball Expansion

Equation (1) and (2) were used to calculation of K_S_ and E_S_, respectively The R_o_/H ratio was 34.89 ± 0.55 (n = 13) in wild-type mice with H = 50 μm [34]. It was 52.37 ± 0.54 (n = 19) and 59.14 ± 0.46 (n = 37) in young and adult D2 mice, respectively, with H = 33 μm [34]. K_S_ and E_S_ were significantly lower in young and adult D2 mice compared to B6 mice, and K_S_ and E_S_ were very close to K_SM_ and E SM in the adult D2 mouse (Table 1), respectively. The data supports a reliable calculation and indicates that D2 mice generally possess a weaker eyeball wall than the wild-type mice.

Previously, patients with a gas bubble injected in vitreous cavity were studied during air flight [35]. Ascending (acute reduction of ATM) and cruising (keeping a high altitude and a low ATM for tens of minutes) phases of the flight were reported to cause distinctive shifts of IOP. Using their data and assuming a V_S_ of 4.96 ml, vitreous space of 4ml, ATM of 760mmHg and gas bubbles obeying Boyle’s law, we calculated that their average E_S_ in vivo was 1.8 × 10^5^ N/m^2^ during cruising phase (n=6). This value was comparable to E_S_ in the wild-type mouse (0.7 × 10^5^ N/m^2^) during long-term physical eyeball expansion. On the same patients, K_S_ at peak IOP during ascending was calculated to be 1.2 × 10^7^ N/m^2^. R/H ratio used for the E_S_ calculation was 10 with the thickness of sclera being taken as H [25, 36, 37]. Such non-invasive artificial modulation of IOP and V_S_ was performed on living patients in a relative shorter period of time, yet a lower elasticity was revealed for the chronic eyeball expansion (cruising phase), in line with the data in the D2 mouse.

### 3.5 Ocular Pressure-Volume Relation

Assuming the volume change of the eyeball content (ΔV_S_) was dominated by the change of the aqueous humor amount (ΔV_W_) for the intact eyeball, then ΔV_S_ ≈ ΔV_W_. Combining this with Equations (1) and (2), it was further deduced that:

(10) $$\Delta \text{IOP}=K_{s}*\Delta V_{w}/V_{s}$$

or

(11) $$\Delta IOP=(2/3)*[1/(1-v)]*(H/R)*E_{s}*\Delta V_{w}/V_{s}\;=1.33*(H/R)*E_{s}*\Delta V_{w}/V_{s}$$

then E_S_ can be expressed as function of K_S_ by substitution in Equation (2),

(12) $$E_{s}=(3/2)*(1-v)*(R/H)*K_{s}=0.75*(R/H)*K_{s}$$

Equations (10) and (11) show that the alteration of IOP is related to at least three factors: positively correlated with the elasticity and negatively correlated with the accumulation of aqueous humor relative to the eyeball volume. Thus, even if aqueous humor increases, a decrease of K_S_ and an increase of V_S_ may buffer IOP change or mask IOP elevation. The equations explain well why certain glaucoma patients and D2 mice with assumed accumulation of aqueous humor did not show elevation of IOP. It was also in alignment with the concept that the accumulation of aqueous humor is an important factor for IOP elevation. The fact that the glaucoma D2 mouse had lower K_S_ and E_S_, bigger V_S_, larger ΔV_W_, but a normal IOP level was also fully accounted for by the equations, supporting the validity of the model.

Moreover, pressure is exerted under physiological conditions against both sides of the eyeball shell and the retina. The outside pressure (P_out_, inward) and inside pressure (P_in_, outward) have to be balanced, i.e. P_in_ = P_out_. For the eyeball shell and the retina, P_out_ = ATM + P_S_ (P_S_, the restoring pressure of the expanded eyeball shell) and P_in_=ATM + IOP (P_in_, presumably contributed primarily by the restoring pressure of the compressed eyeball contents and blood pressure). Thus the total radical stress [38] on the eyeball shell and retina is about *σ*_rr_ = (1/2) * (2ATM + 2IOP). According to Young’s modulus, the radial elasticity of the eyeball shell E_H_ = *σ*_rr_ /ξ_H_, and the strain (ξ_H_, ΔH/H, pressure-related thickness change relative to the original thickness of the eyeball shell) can be calculated as:

(13) $$\Delta \text{H}/\text{H}=(1/2)*(2\text{ATM}+2\text{IOP})/{\text{E}}_{\text{H}}=(\text{ATM}+\text{IOP})/{\text{E}}_{\text{H}}$$

Similarly, the radial elasticity of the retina E_h_ = *σ*_rr_ /ξ_h_, and the strain (ξ_h_, Δh/h, pressure-related thickness change relative to the original thickness of the retina) can be calculated by:

(14) $$\Delta \text{h}/\text{h}=(1/2)*(2\text{ATM}+2\text{IOP})/{\text{E}}_{\text{h}}=(\text{ATM}+\text{IOP})/{\text{E}}_{\text{h}}$$

Equations (13) and (14) express that the opposing-shell forces would theoretically cause thinning of the eyeball shell and the retina; and the strain is negatively correlated with the elasticity and positively correlated with the pressure. Clinical IOP levels typically range between 10~50 mmHg, which is nearly 1.2%~6.6% of ATM (760 mmHg). Thus, IOP represents only a small portion of the pressure that the retina is exposed to, hence, the IOP level has a relatively weak effect on the strain of the eyeball shell and retina. This calculation is consistent with a previous report that the eyeball wall is not significantly thinner in older D2 mice compared to 5-month-old D2 mice [34], though the former tends to develop higher IOP than the latter [39]. The permeability of the eyeball shell to gases and water was not clear and not included in the equations.

## 4. Discussion

### 4.1 A Multi-Factor-Meditated Perturbation of Ocular Pressure-Volume Homeostasis Leads to GC Death in NTG and Other Glaucoma Patients

NTG is characterized by GC death and normal IOP [11, 15, 16]. Currently, there is no animal model reported for NTG, and the cause of GC death in NTG is not clear. D2 mice that develop IOP elevation have been widely used as glaucoma animal model for human secondary angle-closure glaucoma [11, 15, 16]. Although the ocular pathology in the animal is identified as an inherited disorder [39], nearly half of the inbred animals do not show IOP elevation. GC population and retinal structure in the D2 mice with normal IOP have not been systematically examined previously [4, 5, 40, 41]. In this paper, we reported that GC death in the D2 mouse retina could occur without IOP elevation, in agreement with previous findings in NTG [11, 15, 16] and a result from the D2 mouse [42]. The D2 mouse with GC loss but normal IOP resembles human NTG and thus can be considered as an animal model for NTG.

Meanwhile, our data provide novel mechanisms that may mediate GC death in the NTG and other glaucoma patients: low K_S_/E_S_ ↔ V_S_ expansion (increase of ΔV_W_) → retinal damage. All of the above four factors were observed in the adult D2 mouse. Retinal volume is calculated as area * thickness, thus universal eyeball expansion predicts an expansion of the retinal area (x-y expansion) and reduction of the thickness (z-compression), which may directly cause damage on retinal GCs. Since IOP represents only a small portion of the pressure that the retina endures (see results), retinal expansion is likely an important factor mediating GC loss in glaucoma, especially NTG. Furthermore, this chain reaction may not be restricted to NTG. It could be effective for other types of glaucoma patients if V_S_, E_S_ or K_S_ is altered.

The data and Equations (10) and (11) indicated a reciprocal causal relation among IOP, K_S_/E_S_, ΔVw and V_S_. The interactions can directly alter retinal structure with or without changes in IOP. This multi-factor pressure-volume model is applicable for NTG and other types of glaucoma, as NTG appears to be a special case when IOP does not change due to a decrease of K_S_/E_S_ and increases of ΔV_W_ and V_S_. Increased ΔV_W_ causing elevation of IOP that was observed in glaucoma patients is reconcilable with the model, if K_S_ is assumed to be constant or reduces moderately and ΔV_W_/V_S_ increases significantly. To our knowledge, this model is the first model for the pressure-volume homeostasis in the eye.

We did not monitor IOP history. All mice were tested in daytime and under similar experimental conditions. The age-corrected IOP elevation in the D2 mouse was similar to previous reports [4, 39, 40, 42]. But the IOP level that we observed in the adult D2 mouse was close to that in the wild-type mouse. Similar to our results, in a previous physiological study in D2 mice (2~10 months) showed an IOP level below that in the wild type mouse [42]. It is possible that we missed certain IOP peak that a mouse may have for a short period of time, especially during nighttime. However, the IOP measured during nighttime in the mouse and human is only about 3 mmHg higher than during daytime [43, 44], and this difference is expected to be reduced in our results due to the 1~2 hours of dark-adaptation before IOP measurement. We chose D2 mice for the experiments without IOP preference, which might account for the normal IOP level in our results.

### 4.2 Low Elasticity of the Eyeball Shell could Possibly Initiate Glaucoma

The lowered elasticity of the eyeball shell could be more vulnerable to the stretch caused by the accumulation of aqueous humor. In humans, E_s_ was 2.45×10^4^ N/m^2^ S measured in cornea in vivo [27], 6.0 × 10^5^ N/m2 in strips of choroidal complex and 1.8~2.9 × 10^6^ N/m^2^ in sclera strips [26]. In pigs, E_S_ was reported to be 0.5~2.4 × 10^5^ N/m and 1.5~8.3×10^5^ N/m for the cornea and sclera, respectively, in freshly isolated intact eyeballs [25]. Taking H as the sclera thickness and thereby the R/H ratio around 10, 52–59, and 35 in the human [25], D2 mouse, and control mouse, respectively [34] (our data), E_S_ that we estimated in the mouse and human was in line with these previous findings.

In the D2 mouse, GC loss was highly correlated with eyeball expansion, while the latter may be contributed, at least partially, by the low E_S_/K_S_. Eyeball enlargement was nearly proportional, indicating expansion of anterior chamber and accumulation of aqueous humor in the D2 mouse, whether primarily or not. This volume change is expected to cause further consequences, e.g. damage to the trabecular meshwork, blood vessels and astrocytes, ischemia and hypoxia, inflammation and immune reaction, etc. Furthermore, in our data, a low E_S_/K_S_ was present in young D2 mice, in which the retinas were just starting to lose GCs. This data and Equations (10) and (11), in conjunction with the clinical finding of eyeball enlargement in childhood glaucoma, demonstrate that low eyeball elasticity is related to multiple symptoms of glaucoma in the D2 mouse, and it is likely an initiative factor for glaucoma. The low E_S_/K_S_ is presumably a function primarily of the sclera, but its biological basis is still to be discovered.

A precise measurement of physiological E_S_/K_S_ of eyeballs needs stable physical conditions, and the pressure and volume changes need to be perfectly repeatable and controlled and measured without interferences. We examined E_S_/K_S_ from intact living eyeballs without artificial manipulation of IOP and V_S_. Hence our data is not affected by artificial damages (due to manually altering IOP and V_S_), the instantaneous modulation of aqueous humor generation and drainage (due to acute alteration of IOP or V_S_) and the loss of physical environments (due to isolation of tissue pieces). However, our E_S_/K_S_ may be influenced by tissue growth and chronic adaptation. H is a parameter that is subjective to these two influential factors. We have included H in E_S_ calculation but H is not significantly different between the elder and 5-month-old D2 mice [34]. To further minimize the influence of the two factors, we used age-matched wild-type mice as controls for young D2 mice, which are assumed to share a similar growth rate and adaptation mechanism. A low elasticity was revealed in the young D2 mouse, and the adult D2 mouse exhibited similar elasticity. Additionally, eyeballs in living human subjects, whose V_S_ and IOP were altered by noninvasive approaches in a relatively shorter period of time (tens of minutes), showed lower elasticity upon chronic expansion compared to acute expansion. Therefore, the low elasticity in the D2 mouse was believed to be genuine.

Eyeballs are expected to be imperfectly elastic. Thus, K_S_ reported here generally presents the resistance of the eyeball to a volume change upon a universal pressure, instead of a capability to recover to its original size after IOP is restored. Because the volume of an intact eyeball was hard to alter manually and frequently without damaging the eyeball, the elastic limitation was not determined.

### 4.3 Mouse Eyeballs Possess Universal Inner Pressure and Expand Homogeneously in the D2 Mouse

The anterior chamber volume could be enlarged in glaucoma due to accumulation of aqueous humor or eyeball expansion. Such enlargement can be reflected by increased height of the cornea spherical cap or by a shallower indentation at the sclerocorneal junction in this mouse model. However, it has not been reported whether the pressure inside the eyeball is homogenous; and correspondingly it is not certain whether an increase of aqueous humor causes universal eyeball expansion or only partially enlargement restricted to the anterior portion of the eye in glaucoma.

The eyeball encloses a continuous cavity with an elastic shell, and its contents are primarily composed of water (i.e. 99.9% for aqueous humor, 99% for vitreous humor and 75% for the lens). Because of this structure and the great bulk modulus of water (K_W_, 2.15 * 10^9^ N/m^2^), theoretically the pressure among the eyeball compartments should be balanced. In accordance with this expectation, we found that eyeballs in the control mouse maintained a nearly perfect spherical shape and that in adult D2 mice, the eyeballs were expanded significantly but only elongated very moderately. The data supported that the pressure was nearly homogenous inside the eyeball, at least for the mouse, and eyeball expansion was homogeneous in the D2 mouse.

### 4.4 V_S_, Eyeball Elasticity, and Aqueous Humor Share the Responsibility on IOP Modulation

Accumulation of aqueous humor has long been believed to result in elevation of IOP, yet it has not been studied how the compressibility of the aqueous humor and the size of the buffering space influence the outcome [20].

The large K_W_ gives water a well-known reputation of being non-compressible, and the aqueous humor is composed of 99.9% of water, it is expected to have a bulk modulus near water. Based on K_W_, a moderate pressure of 770~810 mmHg (ATM 760 mmHg plus IOP 10–50 mmHg) will cause a volume compression of aqueous humor as little as 0.000048%~0.000050% [ΔV_W_/V_W_ = (ATM + IOP)/ K_W_]. Thus, under clinical IOP levels, additional aqueous humor requires additional space, a space near 99.9999% of its uncompressed volume.

Due to the extremely low compressibility and the fluctuation [45–50] of aqueous humor, special mechanisms are required to stabilize IOP. Modulation of aqueous humor circulation is a well-known mechanism for it. Yet it is not necessarily the only mechanism. Given normal eyeball volume in human is about 6.5 ml and the thickness of the sclera is 1mm [36, 37], the inner space of the eyeball is calculated to be 4.96 ml. Assuming that the eyeball shell is not expandable and all eyeball contents are composed of water, every 1 μl of an extra amount of aqueous humor would elevate IOP for 500 mmHg for an eyeball of 4.96 ml. Yet, this hypothetic elevation of IOP never happens despite that the aqueous humor may fluctuate around 1 μl [45–50]. This may be attributed to the elasticity of the eyeball shell and the involvement of the entire ocular space. For the 29-day-old mouse, the eyeball inner space that we calculated based on a previous report was near 14.13 μl, and aqueous humor was approximately 1.98 μl [51]. In our data, the eyeball volume in the mouse was about 5 times the aqueous humor volume. Since V_S_ is about 16 times the chamber volume in the human and 5~7 times for the mouse, additional aqueous humor would cause a relatively moderate IOP elevation if its volume is buffered by V_S_ instead of the chamber space. The trade-off, however, is retinal expansion and neuronal damage if V_S_ changes too dramatically. In our data, eyeball enlargement below 1μl/ month (4%) was tolerated and above 1.9μl per month (10%) was intolerable for GCs.

The volume change of the eyeball shell (ΔV_S_) can be passively caused by an extra amount of aqueous humor (ΔV_W_). It may also be initiated by changes in eyeball elasticity (K_S_/E_S_). It is still unclear how K_S_/E_S_ is modulated and whether muscles atrophy and dysfunction of the nervous system play any role in retinal GC death in glaucoma. However, muscles on the eyeball wall (e.g. ciliary muscle) are likely able to alter V_S_ and K_S_/E_S_ and make them modulated by the nervous system. The ciliary body extends from the ora serrata of the retina to the outer edge of the iris and the sclerocorneal junction [52]. It forms a 3mm band on the outer surface of the choroid between the anterior and posterior of the eyeball wall in the human, and it is innervated by the nervous system. Due to the orientation, its contraction and relaxation may alter Vs and K_S_/E_S_. Hence the ciliary muscle appears to be able to serve as a critical ocular space and pressure modulator, besides its other roles. Studies on V_S_ and K_S_/E_S_ modulation are expected to lead to establishment of novel glaucoma treatments.

In summary, the first animal model resembling human normal-tension glaucoma and a noninvasive approach for measurement of V_S_ and the ocular elasticity were reported. A multi-factor-meditated perturbation of ocular pressure-volume homeostasis is revealed to be a novel potential mechanism to initiate the ganglion cell death in normal-tension glaucoma and other glaucoma patients. Due to the pathological variety of D2 eyes and NTG patients, only a part of D2 retinas could be considered as NTG model, and this study established some primary criteria for this purpose. We defined the relation of those physical factors by the modified bulk modulus and Young’s modulus. To our knowledge, this is the first model to address the ocular pressure-volume relation. The equation could be rearranged in the following 6 forms:

I: $$\Delta \text{IOP}=\text{Ks}*\Delta {\text{V}}_{\text{W}}/{\text{V}}_{\text{S}}$$

II: $$\Delta \text{IOP}=1.33*(\text{H}/\text{R})*{\text{E}}_{\text{S}}*\Delta {\text{V}}_{\text{W}}/{\text{V}}_{\text{S}}$$

III: $${\text{V}}_{\text{S}}={\text{K}}_{\text{S}}*\Delta {\text{V}}_{\text{W}}/\Delta \text{IOP}$$

IV: $${\text{V}}_{\text{S}}=1.33*(\text{H}/\text{R})*{\text{E}}_{\text{S}}*\Delta {\text{V}}_{\text{W}}/\Delta \text{IOP}$$

V: $$\Delta {\text{V}}_{\text{W}}/{\text{V}}_{\text{S}}=\Delta \text{IOP}/{\text{K}}_{\text{S}}$$

VI: $$\Delta {\text{V}}_{\text{W}}/{\text{V}}_{\text{S}}=\Delta \text{IOP}/\left[1.33*(\text{H}/\text{R})*{\text{E}}_{\text{S}}\right]$$

Equation I and II state that IOP alteration is positively correlated with the elasticity and the volume fluctuation of the aqueous humor relative to the eyeball volume; Equation III and IV state that the eyeball volume is positively correlated with the elasticity and the volume fluctuation of the aqueous humor relative to the IOP change; and Equations V and VI state that the eyeball expansion rate is negatively correlated with the eyeball elasticity and positively correlated with the IOP elevation. As IOP contributes only a small portion of the pressure that retina exposes to, eyeball expansion and low elasticity are likely more important factors mediating GC loss in glaucoma, especially in NTG.

## Figures and Tables

**Table 1A:** Simultaneous measurement of IOP and the volume and elasticity of eyeballs.

Strain	age, mean	s.e.m	mean	s.e.m	n	P			
						B_6_:D_2_ 3.6M	B_6_:D_2_ 9.0M	D_2_ 3.6: 9.0 M	
	months		IOP (mmHg)						
B_6_	5.9	0.6	12.45	0.82	30	6.37E-04	0.439	0.010	
D_2_	3.6	0.3	8.77	0.38	22				
D_2_	9.0	0.6	11.67	0.77	42				
			V_S_(μL)						
B_6_	4.4	0.3	22.43	1.09	13	0.620		1.02E-06	
D_2_	3.6	0.3	21.84	0.63	20				
D_2_	9.0	0.6	30.49	1.09	38				
			K_S_(N/m^2^)						K_SM_(N/m^2^)
B_6_	4.4	0.3	3269.97	670.83	12	8.80E-04		0.679	
D_2_	3.6	0.3	1022.93	250.77	19				
D_2_	10.8	0.3	913.47	134.76	38				973.83
			E_S_(N/m^2^)						E_SM_(N/m^2^)
B_6_	4.4	0.3	85767.23	8983.07	12	0.014	0.023	0.859	
D_2_	3.6	0.3	38156.51	9843.84	18				
D_2_	10.8	0.3	40101.08	5912.27	37				43091.98

**Table 1B:** Simultaneous survey of GCs, ACs and total neurons in the GCL.

Strain	age, mean	s.e.m	cells, mean	s.e.m	n	p		Cell loss, %
						B_6_:D_2_ 3.3M	D_2_ 3.3: 10.7 M	
	months		GCs					
B_6_	3.4	0.4	49374	2211	6	0.326	0.049	
D_2_	3.3	0.6	39005	8874	7			
D_2_	10.7	0.7	18024	5568	12			53.8%
			displaced ACs					
B_6_	3.4	0.4	62326	2955	4	0.863	0.006	
D_2_	3.3	0.6	69588	4459	4			
D_2_	10.7	0.7	51838	2871	10			25.5%
			Total neurons in the GCL					
B_6_	3.4	0.4	113223	4340	4	0.476	0.025	
D_2_	3.3	0.6	101503	10808	4			
D_2_	10.7	0.7	67783	6116	10			33.2%

Note: IOP: intraocular pressure. V_S_: eyeball volume. K_S_: volumetric elasticity of the eyeball. E_S_: tensile elasticity of the eyeball shell. K_SM_ and E_SM_: the volumetric and tensile elasticity calculated based on the difference of average IOP and the difference of average volume of the two age groups of D2 mice. B6: C57BL/6J. D2: DBA/2J. GCs-ganglion cells. ACs: amacrine cells. The data show a significant low elasticity and eyeball expansion in the D2 mouse (1A). GCs and total neurons were surveyed simultaneously on the same animals. A significant loss of GCs and ACs are evident in adult D2 mice (1B).

**Table 2:** Measurement of eyeball 3D dimensions in the wild-type and D2 mouse.

Strain	age, mean	s.e.m	mean	s.e.m	n	p			
						B_6_:D_2_ 3.6M	B_6_:D_2_ 10.8M	D_2_ 3.6: 10.8M	
	months		d_x_d_y_ (mm)						d_x_: d_y_: d_z_
B_6_	4.4	0.3	3.47	0.07	11	0.576		7.52E-05	1:1:1
D_2_	3.6	0.3	3.38	0.09	6				1:1:1.044
D_2_	10.8	0.3	3.89	0.05	25				1:1:1.005
			Z_C_ (mm)						V_C_ (μl)
B_6_	4.4	0.3	1.33	0.03	11	0.247	0.207	0.059	6.60
D_2_	3.6	0.3	1.22	0.11	6				5.81
D_2_	10.8	0.3	1.41	0.04	24				8.79
			d_z_ (mm)						
B_6_	4.4	0.3	3.47	0.06	11	0.443		4.13E-04	
D_2_	3.6	0.3	3.53	0.08	6				
D_2_	10.8	0.3	3.91	0.04	18				
			R_o_ (mm)						
B_6_	4.4	0.3	1.75	0.03	12	0.511		8.73E-12	
D_2_	3.6	0.3	1.74	0.02	18				
D_2_	9.0	0.6	1.95	0.02	37				

Note: d_x_, d_y_ and d_z_: the width, height and depth of the eyeball, respectively. Z_C_: the height of the cornea spherical cap. V_C_: the inner volume of cornea spherical cap for estimation of anterior chamber space. R_o_: outer radius of the eyeball. B6: C57BL/6J. D2: DBA/2J. The data reveal a nearly perfect spherical shape in eyeballs of wild-type and D2 mice and the universal expansion in the adult D2 mouse.

**Table 3:** Factors correlated with neuron counts in the GCL in the D2 mouse.

	n(retina)	age	IOP	d_x_, d_y_	Z_C_	d_z_	V_s_
GC counts							
t value	12	−0.7478	−0.5016	−0.8270	−0.9264	−0.9080	−0.8411
P value		0.0052	0.0966	0.0009	< 0.0001	< 0.0001	0.0006
Total neuron counts							
t value	7	−0.8655	−0.5546	−0.8025	−0.9254	−0.9664	−0.8072
p value		0.0118	0.1959	0.0297	0.0028	0.0004	0.0282

Note: d_x_, d_y_ and d_z_: the width, height and depth of the eyeball, respectively. Z_C_: the height of the cornea spherical cap. GC: ganglion cell. GCL-GC layer. IOP: intraocular pressure. Table indicates a close correlation between neuron populations (including GCs and total neurons in the GCL) and the eyeball 3D expansion. The statistical significance of correlation coefficients is d_z_>Z_C_>d_x_/d_y_>V_S_>age>IOP.

## Abbreviation list

V_S_: eyeball volume
ΔV_S_: changes in V_S_
V_W_: the volume of aqueous humor
ΔV_W_: changes in V_W_
IOP: intraocular pressure
ΔIOP: changes in IOP
K_S_ and K_SM_: bulk modulus of the eyeball
K_W_: bulk modulus of water
E_S_ and E_SM_: Young’s modulus of the eyeball shell
ν: Poisson’s ratio
H: the thickness of the eyeball shell
R_i_ or R_o_: inner or outer radius of the eyeball
d_x_, d_y_ and d_z_: the width, height and depth of the eyeball, respectively
Z_C_: height of the cornea spherical cap
Z_R_: height of the spherical cap that the retina covers
V_C_: volume of the cornea spherical cap
α: coverage of the retina in the eyeball
β: coverage of the cornea on the eyeball
ATM: atmospheric pressure
